# Can mood disorder in women with breast cancer be identified preoperatively?

**DOI:** 10.1038/bjc.1995.538

**Published:** 1995-12

**Authors:** A. J. Ramirez, M. A. Richards, S. R. Jarrett, I. S. Fentiman

**Affiliations:** ICRF Clinical Oncology Unit, Guy's Hospital, London, UK.

## Abstract

The Hospital Anxiety and Depression (HAD) scale, a self-report questionnaire, was tested as a method of identifying mood disorder among patients with operable breast cancer during the year after diagnosis. In a cohort of 91 patients anxiety and depression were assessed preoperatively, and at 3 and 12 months post-operatively, using a standardised psychiatric interview and diagnostic rating criteria. The patients also completed the HAD scale at each assessment. Fifty out of 91 (55%) patients were full or borderline cases of depression and/or anxiety at one or more assessment points. Using a receiver operator characteristic curve analysis, the optimum threshold for the preoperative HAD scale total score to identify psychiatric disorder either preoperatively or at 3 and 12 months post-operatively was 11. With this threshold 70% of both full and borderline cases occurring at any of the assessment points were correctly identified. The false-positive rate was 12%. This approach was particularly sensitive to full cases, correctly identifying 90% of them. The potential for the preoperative HAD scale total score to identify mood disorder in the year after diagnosis was influenced by age. Among women aged less than 50 years, a preoperative HAD scale total score > or = 11 provided a highly sensitive indicator of mood disorder (full and borderline cases) at any time in the year after diagnosis (sensitivity = 90%). The false-positive rate was 40%. Among women older than 50 who experienced a mood disorder, only 57% were correctly identified by a HAD scale total score of > or = 11 (sensitivity = 57%). However, the false-positive rate among older women was low (3%). This simple preoperative screening approach can be used to identify patients who have or are at high risk of developing severe mood disorder in the year after diagnosis. The HAD scale is also sensitive to the detection of borderline mood disorder in patients under the age of 50. It is a specific screening tool among patients over 50, but is not sensitive to the detection of borderline mood disorder in this age group.


British Journal of Cancer (1995) 72, 1509–1512
© 1995 Stockton Press All rights reserved 0007–0920/95 $12.00

### Can mood disorder in women with breast cancer be identified preoperatively?

AJ Ramirez[1,2], MA Richards[1], SR Jarrett[1,2] and IS Fentiman[1]

[1]ICRF Clinical Oncology Unit, Guy's Hospital, London SE1 9RT, UK; [2]Division of Psychiatry and Psychology, UMDS, Guy's Hospital, London SE1 9RT, UK.

**Summary** The Hospital Anxiety and Depression (HAD) scale, a self-report questionnaire, was tested as a method of identifying mood disorder among patients with operable breast cancer during the year after diagnosis. In a cohort of 91 patients anxiety and depression were assessed preoperatively, and at 3 and 12 months post-operatively, using a standardised psychiatric interview and diagnostic rating criteria. The patients also completed the HAD scale at each assessment. Fifty out of 91 (55%) patients were full or borderline cases of depression and/or anxiety at one or more assessment points. Using a receiver operator characteristic curve analysis, the optimum threshold for the preoperative HAD scale total score to identify psychiatric disorder either preoperatively or at 3 and 12 months post-operatively was 11. With this threshold 70% of both full and borderline cases occurring at any of the assessment points were correctly identified. The false-positive rate was 12%. This approach was particularly sensitive to full cases, correctly identifying 90% of them. The potential for the preoperative HAD scale total score to identify mood disorder in the year after diagnosis was influenced by age. Among women aged less than 50 years, a preoperative HAD scale total score $\geqslant 11$ provided a highly sensitive indicator of mood disorder (full and borderline cases) at any time in the year after diagnosis (sensitivity = 90%). The false-positive rate was 40%. Among women older than 50 who experienced a mood disorder, only 57% were correctly identified by a HAD scale total score of $\geqslant 11$ (sensitivity = 57%). However, the false-positive rate among older women was low (3%). This simple preoperative screening approach can be used to identify patients who have or are at high risk of developing severe mood disorder in the year after diagnosis. The HAD scale is also sensitive to the detection of borderline mood disorder in patients under the age of 50. It is a specific screening tool among patients over 50, but is not sensitive to the detection of borderline mood disorder in this age group.

**Keywords**: anxiety; depression; identification; breast cancer; Hospital Anxiety and Depression scale

About 25% of patients with early breast cancer experience anxiety and depression at any one time in the year after diagnosis (Dean, 1987; Fallowfield *et al.*, 1990). This includes depressive illnesses and anxiety states as well as borderline mood disorders. During routine medical care only 20–50% of these distressed patients are recognised and referred for appropriate help (Maguire, 1984*a*). This identification rate might be increased by improving the communication skills and psychiatric interview techniques of cancer professionals (Maguire, 1984*b*).

An alternative approach involves screening for mood disorder by asking patients to complete self-report questionnaires at regular intervals. The effectiveness of the Hospital Anxiety and Depression (HAD) scale (Zigmond and Snaith, 1983) in detecting already existing mood disorder has been assessed in a number of different cancer populations (Razavi *et al.*, 1990, 1992; Hopwood *et al.*, 1991; Ibbotson *et al.*, 1994), but it has not been evaluated specifically in patients with early breast cancer. Furthermore, the value of the HAD scale as a predictive screening tool to identify those patients at risk of developing mood disorder has not been studied.

Preoperative detection of breast cancer patients with depression and/or anxiety and prediction of those likely to develop such disorder post-operatively is important if psychosocial care is to be given appropriately and effectively. We therefore assessed the utility of the HAD scale administered once, preoperatively, in defining patients who either have or are likely to develop mood disorder in the year after diagnosis. The performance of the HAD scale used in this novel way was compared with the effectiveness of serial HAD scale measurements in detecting concurrent disorder at intervals during the post-operative year. Emphasis was placed on identifying patients with mood disorder whether it be anxiety and/or depression, rather than on the particular nature of mood disorder, as this can be assessed subsequently when the patient is seen by a mental health professional.

Correspondence: AJ Ramirez
Received 29 September 1994; revised 27 June 1995; accepted 3 July 1995.

### Patients and methods

A consecutive series of women presenting to the Clinical Oncology Unit, Guy's Hospital, with operable breast cancer (T1,2 N0,1, M0) between April 1989 and June 1990 and who were aged less than 70 were eligible to participate in the study. Patients were treated according to policies in operation at Guy's during the study period. Briefly, patients with unifocal tumours measuring less than 4 cm in diameter were offered breast conservation therapy. Modified radical mastectomy was recommended for those with larger tumours. Postmenopausal patients normally received adjuvant tamoxifen. Premenopausal patients with positive axillary nodes usually received adjuvant chemotherapy.

Each patient was informed about the study and those who agreed to participate underwent psychological assessment on three occasions; immediately before operation and at 3 and 12 months after operation. Psychiatric symptoms experienced over the previous month were elicited at each assessment using a shortened version of the Present State Examination (PSE) (Wing *et al.*, 1974). This is a standardised semistructured interview, which was conducted by a research psychologist (SRJ) who was trained in the use of the PSE. Ratings of psychiatric symptoms were discussed at consensus meetings held between PSE trained raters at Guy's Hospital. Diagnoses of anxiety and/or depression were derived using the Bedford College Criteria (Finlay-Jones *et al.*, 1980). These criteria enable patients to be categorised as normal, borderline cases or full cases of anxiety and/or depression. A full case corresponds to a level of severity of psychiatric disorder that would be likely to be seen in a psychiatric outpatient clinic. Patients found to be full cases and about whom the research psychologist was clinically concerned were referred to the Clinical Oncology Unit liaison psychiatry service. In addition any member of the clinical team was able to refer a patient to the psychiatrist if he/she considered this clinically appropriate.

Patients also completed the HAD scale at each assessment. The HAD scale was developed specifically for use in patients with physical disease and excludes somatic symptoms that could be due either to mood disturbance or physical illness and its treatment. The questionnaire enquires about 14 symptoms of mood disturbance over the preceding week. It is made up of seven items about anxiety and seven items about depression. Each item is rated on a scale of 0–3 ranging from 'not at all' to 'very much'. This gives a maximum score of 42. The HAD scale is quick and easy to administer, complete and score. It usually takes a few minutes for patients to fill in and scoring takes approximately the same time.

### Analysis

The sensitivity and specificity of the HAD scale total score was calculated for all possible threshold scores. This was done by looking at:

(1) The preoperative HAD scale total score in relation to mood disorder at any of the three time points (i.e. in detecting preoperative disorder and in predicting disorder at 3 and 12 months).
(2) The HAD scale total score in relation to concurrent mood disorder at all three time points.

Sentitivity represents the proportion of correctly identified cases (number of true positives/number of true positives plus the number of false negatives). Specificity is the proportion of correctly identified non-cases (number of true negatives/number of true negatives plus number of false positives) and hence the false-positive rate is 1 – specificity.

Receiver operating characteristic (ROC) curves (Murphy *et al.*, 1987) were obtained by plotting sensitivity against (1 – specificity) for each possible threshold score looking at:

(1) The preoperative HAD scale total score in relation to mood disorder at any of the three time points.
(2) The HAD scale total score in relation to concurrent psychiatric disorder.

The ROC curve shows the discriminating power of the HAD scale between full/borderline cases and non-cases at every possible threshold. Examination of the ROC curve allows a decision to be made about the optimum threshold for identifying psychiatric disorder. This was chosen as the score which minimises errors, i.e. the sum of the false positives and false negatives.

The positive predictive value was calculated for each optimum threshold. This gives the proportion of high scorers who are true cases (true positives/true positives plus false positives).

### Results

### *Cohort characteristics*

Ninety-one (89%) of 102 consecutive patients who fulfilled the selection criteria were interviewed at all three assessment times. None of the patients invited to participate declined the first interview, seven declined the second interview and four declined the third interview. The HAD scale was completed by 90 of the 91 patients at all three assessments. One of the 91 patients completed the HAD scale at the first two assessments only. The median age was 56 years (range 24–69). Social class was measured according to the Registrar General's 5-fold classification. Sixty-four (70%) patients were either married or co-habiting, whilst four (4%) were single, eight (9%) were divorced and 15 (17%) were widowed. Six (7%) were in social class 1, with 12 (13%), 58 (64%), six (7%) and nine (10%) in classes 2, 3, 4 and 5 respectively. Six patients were offered specific psychological management, to be undertaken by the unit liaison psychiatrist during the study period. Three declined the offer and three experienced a clinically significant reduction in their levels of anxiety and/or depression following intervention.

### *Inter-rater reliability*

Checks of inter-rater reliability were performed on a random sample of 30% of the tape-recorded interviews. Percentage concordance for individual items within the PSE ranged from 70–100% and for whether patients were normal, borderline cases or full cases from 80–100%.

### *Prevalence of mood disorder*

Preoperative levels of mood disorder assessed by PSE were high, 37/91 (41%) being full or borderline cases of depression and/or anxiety. This declined to 29% at 3 months and 25% at 12 months post-operatively. Fifteen (17%) of the women were full or borderline cases at the preoperative assessment only, 22 (24%) had this level of mood disorder both preoperatively and post-operatively (i.e. at 3 months, 12 months or both). Thirteen (14%) developed full or borderline case level anxiety and/or depression post-operatively. Fifteen (17%) of the women were full cases preoperatively; seven (8%) at 3 months and one (1%) at 12 months. A total of 20 (22%) patients were classified as full cases at one or more assessments. A further 30 (33%) patients had borderline anxiety and/or depression on one or more occasions but were never full cases. The remaining 41 (45%) patients had no significant anxiety or depression at any of the three assessments.

Whilst the prevalence of mood disorder overall decreased progressively over the study period, the patterns of anxiety and depression differed substantially. Eighteen of the 34 patients (53%) who were anxious preoperatively had significant levels of anxiety either at 3 months or 12 months post-operatively, or both. Onset of anxiety post-operatively was unusual in patients who had not had clinically significant levels of anxiety preoperatively, occurring in only six cases (four of whom were either also depressed post-operatively or had been preoperatively). In contrast, only five of the 20 (25%) patients who were depressed preoperatively continued to be depressed post-operatively, but 15 patients developed a depression for the first time post-operatively (seven of whom had either concomitant or preceding anxiety).

Mood disorder was to an extent associated with age. Twenty out of 30 (67%) of patients aged less than 50 were borderline or full cases at any time point, compared with 30/61 (49%) patients aged $\geqslant 50$ ($P = 0.1$). Twelve out of 30 (40%) patients aged less than 50 were full cases, while 8/61 (13%) aged $\geqslant 50$ were full cases ($P = 0.006$).

### *Performance of HAD scale total score*

The potential of the preoperative HAD scale total score to identify patients who experienced mood disorder (full and borderline cases) at any time in the year after diagnosis was examined. This involved assessing the ability of the preoperative HAD scale score to detect current mood disorder and to predict disorder at 3 and 12 months post-operatively. Adopting this approach the ROC curve demonstrated that the optimum threshold was 11 for the HAD scale total score. Using this threshold, 31/37 (84%) patients who were borderline or full cases of anxiety or depression preoperatively were correctly identified, with a false-positive rate of 9/54 (17%). In addition, 19/26 (73%) patients with mood disorder at 3 months and 19 of 23 (83%) patients with mood disorder at 12 months had a preoperative HAD scale total score $\geqslant 11$. Four out of 13 (31%) patients who developed mood disorder for the first time either at the 3 month or 12 month assessment had a preoperative HAD scale total score $\geqslant 11$. Using this cut-off overall, however, 35 out of the 50 women who had mood disorder (full and borderline cases) at some point in the year after diagnosis were correctly identified (sensitivity = 70%). Five out of 41 women who never experienced mood disorder were incorrectly identified (false-positive rate = 12%). Thirty-five out of 40 of those scoring ≥11 were true full or borderline cases (positive predictive value = 88%). The preoperative HAD scale total score was particularly sensitive to full cases, identifying 18/20 (90%) of such patients.

We examined whether the use of serial HAD scale measurements (at 0, 3 and 12 months) to detect concurrent mood disorder could improve on the identification rate described above, using a single preoperative assessment (both for detection and prediction). First we applied a uniform cut-off of HAD scale total score ≥11 at each of the three time points. Using this method 31 patients were identified correctly preoperatively (see above). In addition one patient with late onset mood disorder was identified at 3 months. Thus, in total, 32 of the 50 patients who had mood disorder at one or more time points were identified using this approach (sensitivity = 64%).

A further analysis of the utility of serial HAD scale measurements for the detection of concurrent mood disorder was undertaken using optimum cut-off levels derived from ROC curve analyses for each time point. At 3 months the optimum total HAD scale score was five (sensitivity = 77%; specificity = 52%; positive predictive value = 38%). The optimum total HAD scale score at 12 months was six (sensitivity = 77%; specificity = 66%; positive predictive value = 40%). These cut-off points are, of course, well below those usually applied using the HAD scale. Applying these optimum cut-offs to detect late-onset mood disorder, seven patients who developed anxiety or depression at 3 months were identified and four patients who developed mood disorder at 12 months were identified. Combining the use of preoperative HAD scale total score ≥11 to identify cases at that time point with the use of the HAD scale total scores using optimum cut-offs at 3 and 12 months, 42/50 cases were identified. However, using this approach, 29 out of 41 who never had mood disorder were incorrectly identified (false positive = 71%).

*Effect of age on performance of preoperative HAD scale total score*

Preoperative HAD scale total scores and age were highly significantly inter-related. Twenty-two of the 30 (73%) patients aged less than 50 years had preoperative HAD scale total scores ≥11, compared with 18 of 61 (30%) of patients above this age ($P = 0.0001$). The potential of the preoperative HAD scale total score to identify psychiatric disorder in the year after diagnosis was influenced by age.

A HAD scale total score measured preoperatively provided an effective indication of mood disorder occurring at the time or at 3 and 12 months post-operatively in patients aged less than 50 (Figure 1). Eighteen of the 22 patients aged less than 50 years who had preoperative total HAD scale score ≥11 experienced mood disorder at some point in the year after diagnosis (positive predictive value = 82%). A preoperative HAD scale score ≥11 correctly identified 18 of the 20 patients aged less than 50 years who had mood disorder (full or borderline cases) at any time point during the study (sensitivity = 90%). The remaining two patients had borderline depression at one assessment point only. This threshold score correctly identified all 12 patients aged less than 50 years who were full cases of anxiety or depression at any time point (sensitivity = 100%). Four out of ten patients who never experienced mood disorder were incorrectly classified (false-positive rate = 40%).

Amongst older patients a different pattern was observed. Seventeen out of 18 patients older than 50 who had a preoperative total HAD scale score ≥11 experienced mood disorder at some point in the year after diagnosis (positive predictive value = 94%). However, only 17/30 patients in this age group who experienced mood disorder were correctly identified by HAD scale total score ≥11 (sensitivity = 57%).

**Figure 1** Distribution of preoperative HAD scale total score according to experience of mood disorder in the year after diagnosis. □, normal; +, borderline; *, full cases. HAD scale total score of 11 is the cut-off point.

Thirty of 31 patients who had no mood disorder during the study period had total HAD scale scores of <11 (false-positive rate = 3%). Lowering the cut-off for the total HAD score improved sensitivity only slightly, but increased the false-positive rate substantially.

The low sensitivity of the preoperative total HAD scale score ≥11 in the older group was mainly in relation to borderline cases. Six out of eight patients older than 50 who were full cases were correctly identified (sensitivity = 75%).

### Discussion

The prevalence of mood disorder experienced at one or more assessment points in the first year after diagnosis of operable breast cancer in this study is high (22% of patients were full cases and 33% were borderline cases at some point). The point prevalence of mood disorder at each of the three assessment points (41% preoperatively, 29% at 3 months and 25% at 12 months post-operatively) is similar to those reported in other studies, despite the use of somewhat different criteria to define psychiatric illness (Dean, 1987; Fallowfield *et al.*, 1990). Psychiatric interventions provided during the study by either the unit liaison psychiatrist or general practitioner are therefore unlikely to have affected significantly the prevalence of mood disorder reported in this study. In any case, only a very small number of patients received treatment from the unit psychiatrist during the study period. Details of interventions initiated or given by a general practitioner were not sought in this study.

The performance of a single HAD scale total score measured preoperatively in identifying mood disorder occurring at any time in the year after diagnosis was judged to be superior to other approaches using the HAD scale to detect concurrent mood disorder at repeated intervals throughout the year. Administering the HAD scale to identify all cases preoperatively in combination with the HAD scale at 3 and 12 months using optimum cut-offs derived for ROC curve analysis to detect late-onset cases correctly identified more of the women who were anxious or depressed. However, the high proportion of well women who were incorrectly identified, together with the need to administer the HAD

scale on repeated occasions limits the practical utility of this approach in a clinical setting. Similarly the conventional method of using the HAD scale only to detect already existing disorder yields a high false-positive rate, involves repeated administration and lacks the advantage of being able to identify those at risk. The effectiveness of single HAD scale total score in identifying mood disorder in the year after diagnosis lies mainly in its ability to detect early transient mood disorder (i.e. those women who were cases at the preoperative assessment only) and in its ability to detect women whose mood disorder developed early and was sustained (i.e. those with mood disorder both preoperatively and post-operatively).

Among patients aged less than 50, a single HAD scale total score measured preoperatively was an effective indicator of both severe and borderline mood disorder occurring at the time it was performed or during the following year. The findings of this study suggest a simple management policy of administering the HAD scale preoperatively and applying a cut-off of 11 to the total score to identify all younger patients who have or are likely to develop severe mood disorder and 90% of those who are at risk of less severe disorder. From our results 73% of women aged less than 50 will be defined as being at risk. These patients should be interviewed by a specialist nurse trained to elicit psychiatric symptoms using a structured interview. In our series 17 of 22 (77%) women so identified had preoperative mood disorder and these women should be offered appropriate psychological care. Among the remaining five women, one subsequently developed psychiatric disorder. We would therefore recommend that women with a preoperative total HAD scale score $\geqslant 11$ but who are not anxious or depressed at interview (the false positives) should be considered at risk. They should be reassessed by the nurse specialist at least once post-operatively (possibly at 3 months).

Among patients over 50 years, a preoperative total HAD scale score was a highly specific screening tool. It was sensitive for the identification of severe mood disorder but not borderline mood disorder. Slightly less than half of older women who experienced mood disorder according to interview assessment were not high scorers on the HAD scale. This suggests that older women tended not to disclose their psychosocial distress on self-report measures. Whatever the reason, all older patients require regular interview assessment throughout the year after diagnosis (for example 3 and 12 months), if borderline and full case mood disorder is to be diagnosed.

We now routinely record patients' total HAD scale scores shortly after diagnosis. This can alert clinicians to patients who have or are at high risk of developing mood disorder. However clinicians should not be falsely reassured by low scores in women over 50 years. Patients are also invited to make contact with the specialist nurse should they develop psychological difficulties at any time.

Women who are found to be full cases of anxiety or depression at interview with the specialist nurse are likely to benefit from structured psychological therapy (e.g. Greer *et al.*, 1992) either alone or in conjunction with psychotropic medication (Costa *et al.*, 1985). Therefore if they are motivated to receive psychological help this could be initiated by an appropriately trained specialist nurse. Alternatively the nurse could refer them to a psychiatrist or clinical psychologist or liaise with the general practitioner. Women with borderline levels of anxiety and depression are likely to have more diverse needs. Some may benefit from a formal psychological therapy, others from the specialist nurse teaching them self-help techniques for managing anxiety (Snaith, 1992). It is likely that a considerable proportion experiencing borderline mood disorder preoperatively will require little more than honest, but compassionate explanation and reassurance about their disease and its management from the doctors and nurses responsible for their care.

### References

COSTA D, MOGOS I AND TOMA T. (1985). Efficacy and safety of mianserin in the treatment of depression of women with cancer. *Acta Psych. Scand.*, **72**, 85–92.

DEAN C. (1987). Psychiatric morbidity following mastectomy: preoperative predictors and types of illness. *J. Psychosom. Res.*, **31**, 385–392.

FALLOWFIELD L, HALL A, MAGUIRE G AND BAUM M. (1990). Psychological outcome of different treatment policies in women with early breast cancer outside a clinical trial. *Br. Med. J.*, **301**, 575–580.

FINLAY-JONES R, BROWN G, DUNCAN-JONES P, HARRIS T, MURPHY E AND PRUDO R. (1980). Depression and anxiety in the community: replicating the diagnosis of a case. *Psychol. Med.*, **10**, 445–454.

GREER S, MOOREY S, BARUCH JDR, WATSON M, ROBERTSON BM, MASON A, ROWDEN L, LAW MG AND BLISS JM. (1992). Adjuvant psychological therapy for patients with cancer: a prospective randomised trial. *Br. Med. J.*, **304**, 675–680.

HOPWOOD P, HOWELL A AND MAGUIRE P. (1991). Screening for psychiatric morbidity in patients with advanced breast cancer: validation of two self-report questionnaires. *Br. J. Cancer*, **64**, 353–356.

IBBOTSON T, MAGUIRE P, SELBY P, PRIESTMAN T AND WALLACE L. (1994). Screening for anxiety and depression in cancer patients: the effects of disease and treatment. *Eur. J. Cancer*, **30A**, 37–40.

MAGUIRE P. (1984*a*). Communication and patient care. In *Health and Human Behaviour*, Steptoe A and Matthews A. (eds.) pp. 153–173. Academic Press: London.

MAGUIRE P. (1984*b*). The recognition and treatment of affective disorder in cancer patients. *Int. Rev. Appl. Psychol.*, **33**, 479–491.

MURPHY J, BERWICK D, WEINSTEIN M, BORYS GF, BUDMAN SH AND KLERMAN GL. (1987). Performance of screening and diagnostic tests: application of receiver operating characteristic analysis. *Arch. Gen. Psychiatr.*, **44**, 550–555.

RAZAVI D, DELVAUX, N, FARVACQUES C AND ROBAYE E. (1990). Screening for adjustment disorders and major depressive disorders in cancer in-patients. *Br. J. Psychiatr.*, **156**, 79–83.

RAZAVI D, DELVAUX N, BREDART A, PAESMANS M, DEBUSSCHER L, BRON D AND STRYCKMANS P. (1992). Screening for psychiatric disorders in a lymphoma outpatient population. *Eur. J. Cancer*, **28A**, 1869–1872.

SNAITH P. (1992). Psychological treatments for patients with cancer. *Br. Med. J.*, **304**, 1569.

WING J, COOPER J AND SARTORIUS N. (1974). *Measurement and Classification of Psychiatric Symptoms*. Cambridge University Press: Cambridge.

ZIGMOND A AND SNAITH R. (1983). The Hospital Anxiety and Depression Scale. *Acta Psych. Scand.*, **67**, 361–370.


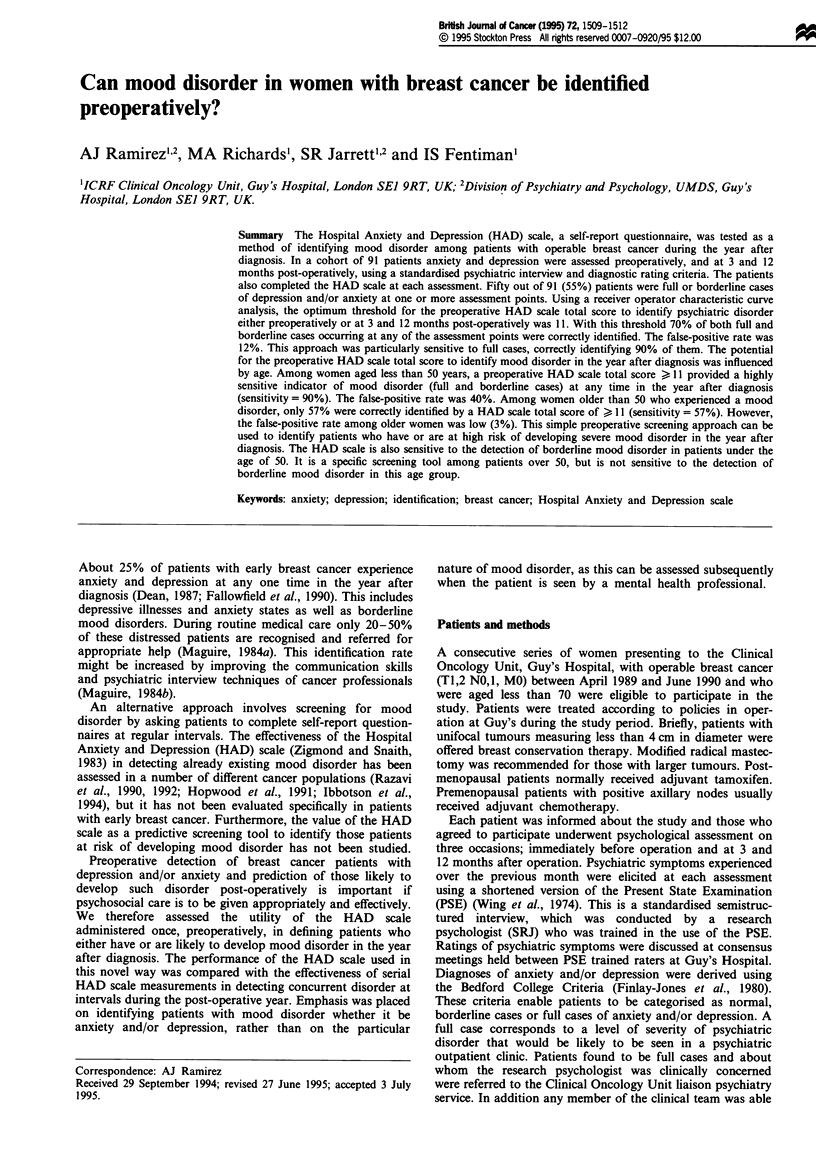

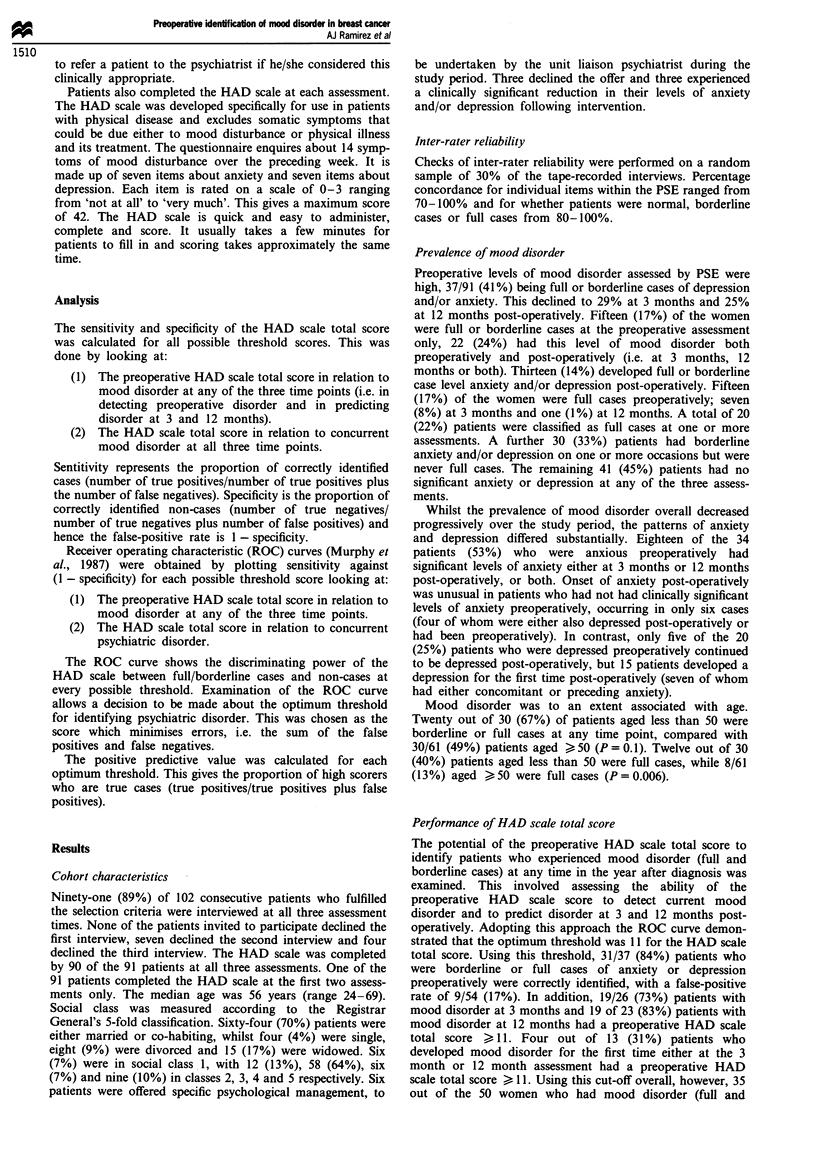

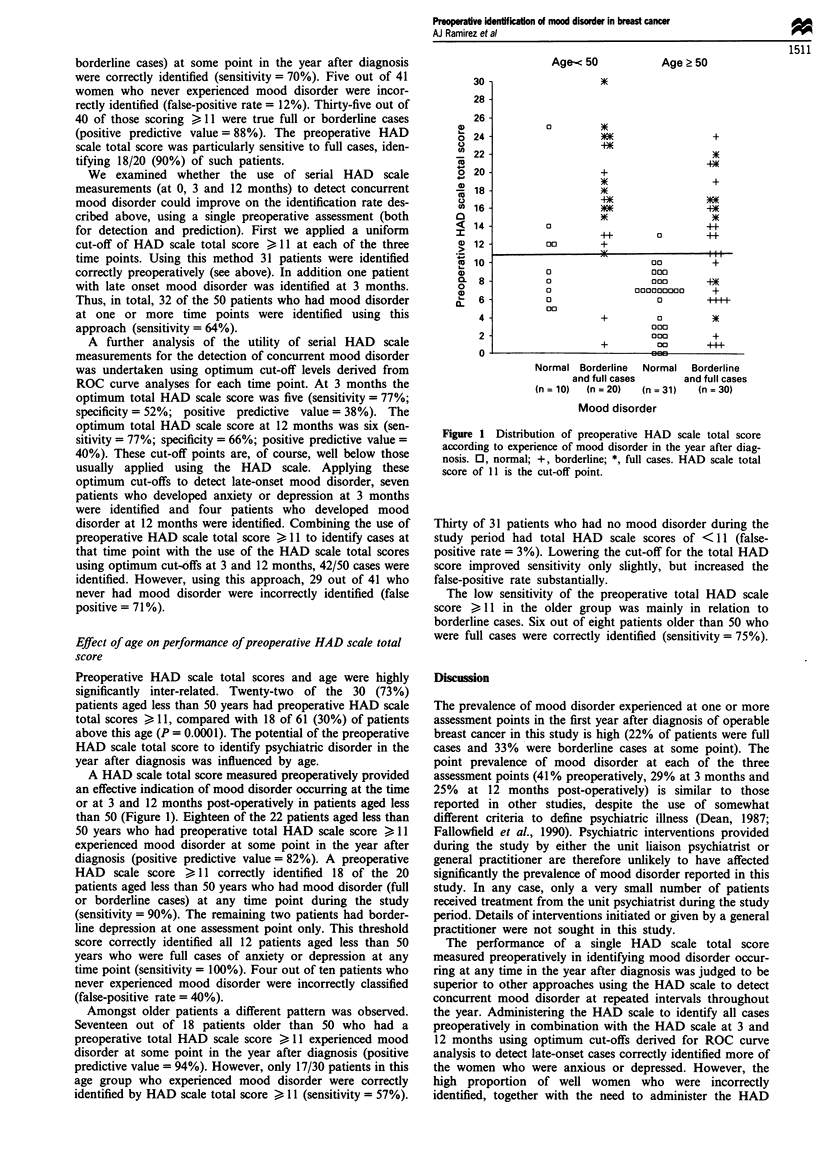

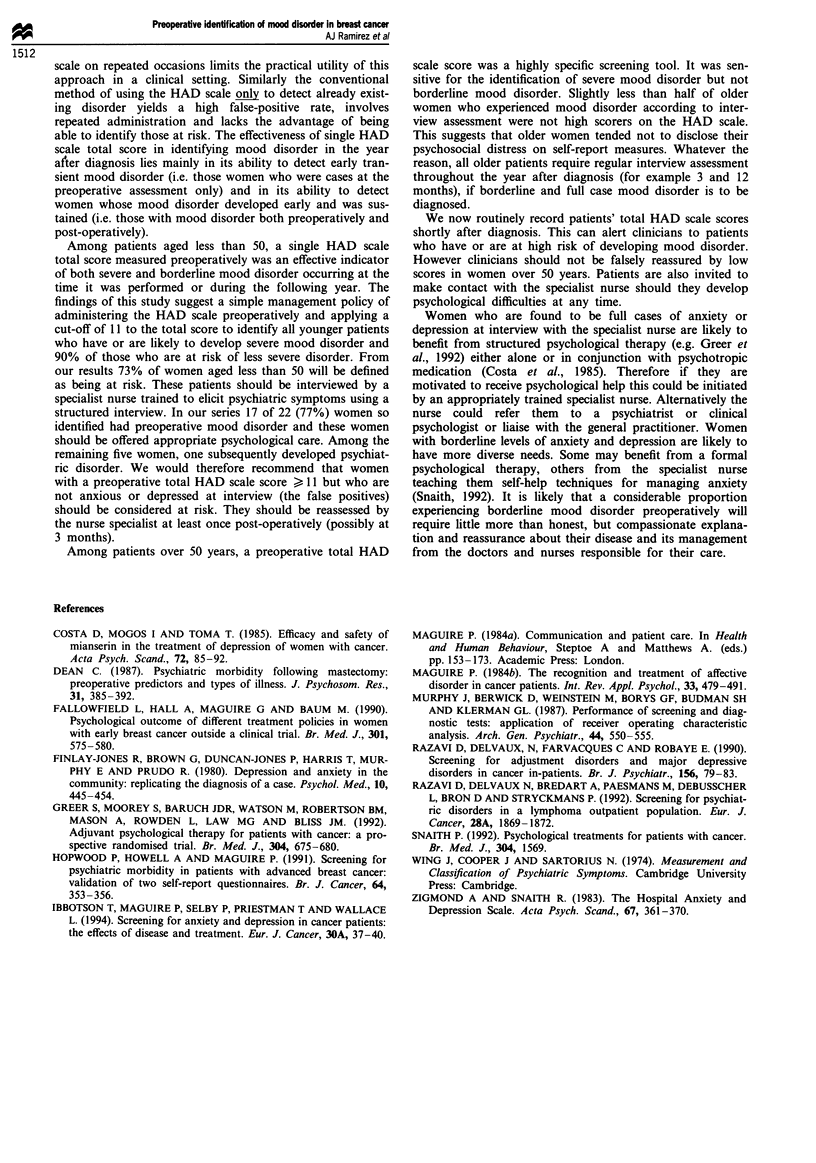


## References

[OCR_00615] Costa D., Mogos I., Toma T. (1985). Efficacy and safety of mianserin in the treatment of depression of women with cancer.. Acta Psychiatr Scand Suppl.

[OCR_00622] Dean C. (1987). Psychiatric morbidity following mastectomy: preoperative predictors and types of illness.. J Psychosom Res.

[OCR_00627] Fallowfield L. J., Hall A., Maguire G. P., Baum M. (1990). Psychological outcomes of different treatment policies in women with early breast cancer outside a clinical trial.. BMJ.

[OCR_00633] Finlay-Jones R., Brown G. W., Duncan-Jones P., Harris T., Murphy E., Prudo R. (1980). Depression and anxiety in the community: replicating the diagnosis of a case.. Psychol Med.

[OCR_00640] Greer S., Moorey S., Baruch J. D., Watson M., Robertson B. M., Mason A., Rowden L., Law M. G., Bliss J. M. (1992). Adjuvant psychological therapy for patients with cancer: a prospective randomised trial.. BMJ.

[OCR_00643] Hopwood P., Howell A., Maguire P. (1991). Screening for psychiatric morbidity in patients with advanced breast cancer: validation of two self-report questionnaires.. Br J Cancer.

[OCR_00651] Ibbotson T., Maguire P., Selby P., Priestman T., Wallace L. (1994). Screening for anxiety and depression in cancer patients: the effects of disease and treatment.. Eur J Cancer.

[OCR_00665] Murphy J. M., Berwick D. M., Weinstein M. C., Borus J. F., Budman S. H., Klerman G. L. (1987). Performance of screening and diagnostic tests. Application of receiver operating characteristic analysis.. Arch Gen Psychiatry.

[OCR_00676] Razavi D., Delvaux N., Bredart A., Paesmans M., Debusscher L., Bron D., Stryckmans P. (1992). Screening for psychiatric disorders in a lymphoma out-patient population.. Eur J Cancer.

[OCR_00670] Razavi D., Delvaux N., Farvacques C., Robaye E. (1990). Screening for adjustment disorders and major depressive disorders in cancer in-patients.. Br J Psychiatry.

[OCR_00679] Snaith P. (1992). Psychological treatments for patients with cancer.. BMJ.

[OCR_00688] Zigmond A. S., Snaith R. P. (1983). The hospital anxiety and depression scale.. Acta Psychiatr Scand.

